# Spontaneous Cultural Conversion Rate of *Mycobacterium avium* Complex Pulmonary Disease Based on BACES Severity

**DOI:** 10.3390/jcm12227125

**Published:** 2023-11-16

**Authors:** Bo-Guen Kim, Jin Young Yu, Byung Woo Jhun

**Affiliations:** 1 Division of Pulmonary Medicine and Allergy, Department of Internal Medicine, Hanyang University College of Medicine, Seoul 04763, Republic of Korea; kbg1q2w3e@gmail.com; 2 Department of Medicine, Samsung Medical Center, Sungkyunkwan University School of Medicine, Seoul 06351, Republic of Korea; jinyoungwlsdud.yu@samsung.com; 3 Division of Pulmonary and Critical Care Medicine, Department of Medicine, Samsung Medical Center, Sungkyunkwan University School of Medicine, Seoul 06351, Republic of Korea

**Keywords:** *Mycobacterium avium* complex, outcome, conversion, prognosis, severity

## Abstract

Background: Only a few clinical factors can aid in predicting spontaneous culture conversion (SCC) in patients with *Mycobacterium avium* complex-pulmonary disease (MAC-PD). In this study, we aimed to evaluate whether the rate of SCC varies according to the severity of the disease in MAC-PD patients. Methods: We retrospectively classified 373 MAC-PD patients who had undergone watchful waiting without antibiotics based on the severity assessment using the ‘body mass index (BMI), age, cavity, erythrocyte sedimentation rate (ESR), and sex (BACES)’ criteria. We evaluated the rate of SCC in MAC-PD patients based on BACES severity and analyzed the relevant factors. Results**:** Of 373 patients, 153 (41%) achieved SCC without antibiotics during a median follow-up of 48.1 months. There was a trend toward a higher SCC rate in patients with lower BACES severity: 48% (87/183), 37% (58/157), and 24% (8/33) in the mild, moderate, and severe BACES groups, respectively. In addition, a favorable outcome, defined as maintaining SCC or having two consecutive negative sputum cultures until the last follow-up date, was also more common in patients with lower BACES severities of 53% (97/183), 34% (54/157), and 18% (6/33) in the mild, moderate, and severe BACES groups, respectively. In multivariate analysis, moderate BACES (hazard ratio [HR] = 0.63; 95% confidence interval [CI] 0.44–0.91; *p* = 0.013) and severe BACES (HR 0.37; 95% CI 0.16–0.90; *p* = 0.028) had a significantly negative impact on favorable outcomes compared to mild BACES. Conclusions: Lower BACES severity may be associated with SCC in MAC-PD patients.

## 1. Introduction

Nontuberculous mycobacteria (NTM) are ubiquitous organisms that cause chronic pulmonary disease (PD), and the burden of this disease is increasing globally [1]. Of more than 200 NTM species, *Mycobacterium avium* complex (MAC), which includes mainly *M. avium* and *M. intracellulare*, is the most common pathogen in many countries [2,3]. While MAC is ubiquitously present, not all individuals exposed to it develop NTM-PD because MAC-PD is a multifactorial disease that occurs due to a complex interaction of environmental factors and the patient’s immune status. For the treatment of MAC-PD, recent guidelines recommend long-term multi-drug antibiotic therapy, including a macrolide, ethambutol, and rifamycin, either with or without injectable aminoglycoside. Additionally, it is recommended to continue antibiotic therapy for at least 12 months, even after successful culture conversion [3,4]. Inevitably, a significant number of MAC-PD patients experience drug-associated adverse effects, such as hepatotoxicity, nephrotoxicity, or visual impairment, due to prolonged antibiotic therapy. Moreover, in patients who do not respond to antibiotic treatment, adjunctive lung surgery is occasionally performed. However, despite aggressive treatment, the long-term treatment success rate remains at 60–70%. Therefore, MAC-PD imposes a significant medical burden on both patients and clinicians [5].

Notably, MAC-PD patients experience a heterogeneous clinical course, unlike other respiratory infectious diseases such as tuberculosis. In approximately 30–40% of patients diagnosed with MAC-PD, the disease does not progress and spontaneous culture conversion (SCC) can occur during the course of observation without antibiotic treatment [6,7]. Therefore, considering the medical burden and adverse effects associated with long-term antibiotic use, as well as the relatively low treatment success rate of antibiotic therapy, guidelines consistently recommend ‘watchful waiting’ instead of immediately starting antibiotic treatment for less severe patients, even after confirming the diagnosis of MAC-PD.

In this context, evaluating clinical factors associated with SCC or those that can predict SCC accurately is crucial in managing MAC-PD patients in real-world clinical practice. This is because accurate predictive factors can assist in determining whether to initiate antibiotic use after the diagnosis of MAC-PD. Unfortunately, there have been no validated prediction tools that can accurately predict SCC in MAC-PD. Although one previous cohort study reported clinical factors related to SCC, such as body mass index (BMI) [6], other recent studies have failed to identify any obvious clinical factors that can predict SCC in MAC-PD patients [7,8]. In addition, previous studies did not reflect the severity of NTM-PD and only analyzed individual factors.

Recently, our research group evaluated the prognostic factors of NTM-PD through the analysis of a study cohort with long-term follow-up data. Subsequently, we developed a disease severity scoring system known as BACES [BMI, age, cavity, erythrocyte sedimentation rate (ESR), and sex], which categorizes the disease into three groups: mild, moderate, and severe [9]. In addition, it has been reported that this BACES severity system also aids in predicting the treatment response in MAC-PD [10,11]. Therefore, in this study, we retrospectively classified MAC-PD patients who underwent watchful waiting without antibiotics based on BACES severity and investigated whether the rate of SCC differed according to BACES severity.

## 2. Methods

### 2.1. Study Design and Population

**Figure 1:**
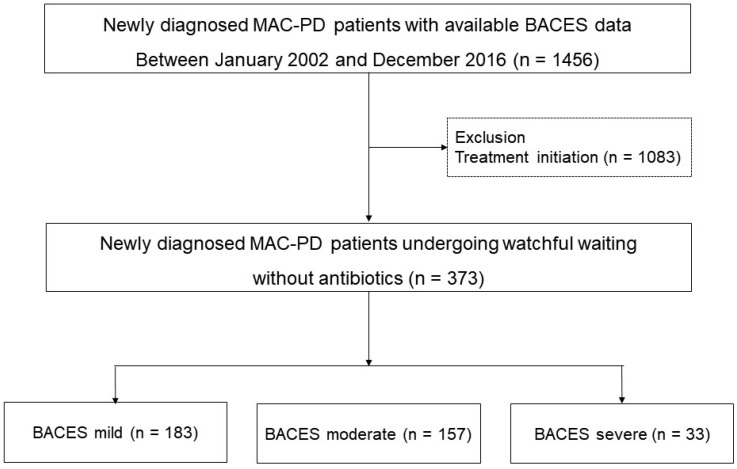
Study population.

Patients who were newly diagnosed with MAC-PD between January 2002 and December 2016 were identified from the NTM Registry of Samsung Medical Center, a referral hospital in Seoul, South Korea. Data were obtained from a retrospective cohort from January 2002 to December 2007 and from an ongoing Institutional Review Board-approved prospective observational cohort study beginning in January 2008 (ClinicalTrials.gov identifier: NCT00970801) [12]. During the study periods, a total of 1456 MAC-PD patients with available BACES severity data were identified. From these screened patients, 1083 patients who started antibiotic treatment due to clinical or radiological deterioration were excluded from the analysis. Finally, in this study, we analyzed 373 newly diagnosed MAC-PD patients who underwent watchful waiting without antibiotics (Figure 1). We investigated the SCC rate of MAC-PD based on the severity of BACES and assessed factors related to SCC using multivariate analysis. Data on clinical outcomes were last updated in June 2021.

### 2.2. Evaluation of BACES Severity

The severity of MAC-PD was determined using the BACES score as follows: BMI < 18.5 kg/m^2^, age ≥ 65 years, presence of a cavity, elevated ESR (men > 15 mm/h and women > 20 mm/h), and male sex. One point was assigned for each item, and the total score was considered an indicator of mild (0–1 point), moderate (2–3 points), or severe (4–5 points) disease [9]. All patients were classified into three groups based on the severity score at the time of diagnosis.

### 2.3. Sputum and Radiological Examinations

At our institution, expectorated sputum was examined at intervals of 3 to 6 months in MAC-PD patients who were observed without antibiotic therapy. Acid-fast bacilli (AFB) smears and cultures of lower respiratory tract samples were obtained using standard methods. Specimens were cultured on 3% Ogawa solid medium (Shinyang, Seoul, Republic of Korea) and in a liquid broth medium in mycobacterial growth indicator tubes (Becton, Dickinson, and Co., Sparks, MD, USA). NTM species were identified using polymerase chain reaction-restriction fragment length polymorphism analysis or reverse-blot hybridization of the rpoB gene. Species were identified via nested multiplex PCR and using a reverse-hybridization assay (AdvanSure™ Mycobacteria GenoBlot Assay; LG Life Sciences, Seoul, Republic of Korea) of the internal transcribed spacer region beginning in June 2014 [13,14].

The radiological phenotype of MAC-PD was classified as either fibrocavitary or nodular bronchiectatic form based on the main features observed on chest computed tomography [12]. The fibrocavitary form was defined by the presence of cavitary opacities and pleural thickening, mainly in the upper lobes. The nodular bronchiectatic form was defined by the presence of multifocal bronchiectasis and clusters of small nodules, regardless of the presence of small cavities.

### 2.4. Evaluation of Microbiological Outcomes

We investigated the rate of SCC in MAC-PD patients who underwent clinical observation without antibiotics during the study period. ‘Culture conversion’ was defined, based on the modified NTM-NET consensus statement, as at least three consecutive negative sputum cultures, collected at least 4 weeks apart [15]. The time to culture conversion was defined as the time to the date of the first negative culture. Most of our study patients had a stable condition with relatively mild respiratory symptoms, and some of them were unable to expectorate sputum for testing during the study periods. Therefore, in this study, for the purpose of analysis, ‘favorable outcome’ was defined as the maintenance of culture conversion or having two consecutive negative sputum cultures without any further positive culture results until the last follow-up date. Time to favorable outcome was defined as the time to the date of the first negative culture in patients in whom a ‘favorable outcome’ was confirmed.

### 2.5. Statistical Analysis

Data are presented as numbers (percentages) for categorical variables and medians (interquartile range [IQR]) for continuous variables. Continuous data were compared using the Mann–Whitney or Kruskal–Wallis test, and categorical data were compared using the chi-square or Fisher’s exact test. Bonferroni’s method was used for post hoc analysis. The Kaplan–Meier method was used to estimate the cumulative conversion or favorable outcome rate, and the log-rank test was used to compare the curves. Cox proportional hazard regression analysis was used to identify factors associated with microbiological outcomes. All tests were two-sided, and a *p*-value < 0.05 was considered significant. All statistical analyses were performed using SPSS Statistics ver. 27 (IBM, Chicago, IL, USA).

## 3. Results

### 3.1. Baseline Characteristics of Study Patients

The baseline characteristics of the 373 MAC-PD patients who underwent watchful waiting without antibiotics are presented in Table 1. Among the patients, 39% were male and 26% had a history of smoking. Twenty percent of patients had a low BMI, and 41% of patients were elderly, being aged 65 or older. The most common underlying condition was previous tuberculosis (28%), followed by chronic obstructive pulmonary disease (6%). More than 60% of patients had a cough or sputum, and 17% of patients had hemoptysis. *M. avium* (61%) was the most common etiology, and the nodular bronchiectatic form was present in 94% of the patients.

The median follow-up period for all study patients was 48.1 (IQR 26.4–76.4) months. Out of the 373 patients, SCC occurred in 153 (41%) of MAC-PD patients. Patients who achieved SCC tended to be relatively young (≤65 years old, *p* = 0.001), have no cavities (*p* = 0.040), and exhibit negative sputum AFB smear results (*p* = 0.024) compared to those with persistent NTM positivity in their sputum test results.

### 3.2. Microbiological Outcomes in Patients without Antibiotic Treatment

Table 2 shows the microbiological outcomes of patients who underwent watchful waiting without antibiotic treatment. During the study periods, SCC occurred in 153 (41%) of all 373 study patients, with a median time to conversion of 6.2 (IQR 2.7–17.7) months. Based on BACES severity, 183 patients had mild disease, 157 had moderate disease, and the remaining 33 had severe disease.

There was a trend toward higher rates of SCC in MAC-PD patients with lower BACES severity. Among mild patients, 48% achieved SCC within a median observation period of 6.0 months, while 37% of moderate disease patients had SCC within a median of 5.4 months. In cases of severe disease, only 24% of patients achieved SCC within a median observation period of 22.1 months. In the cumulative conversion rates evaluated using Kaplan–Meier curves, although not statistically significant, there was a tendency for SCC to occur earlier and more frequently as the BACES severity decreased (Supplementary Figure S1).

**Figure 2:**
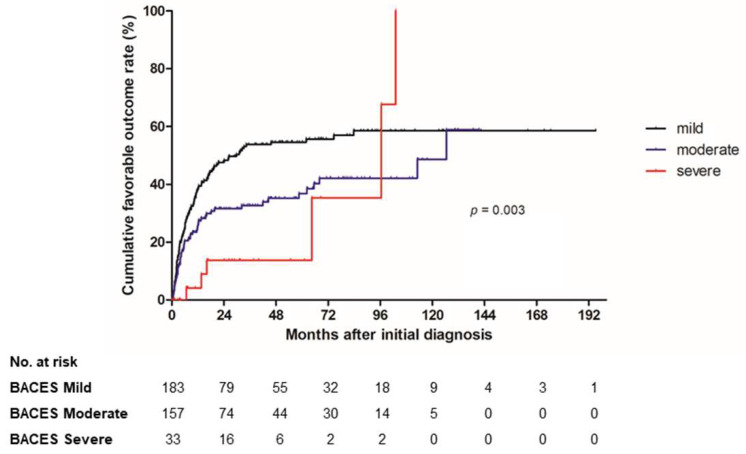
Cumulative favorable outcome rate in all study patients according to BACES severity.

In terms of favorable outcomes, there was a tendency toward a higher rate of favorable outcomes with a decrease in BACES severity. In the mild disease group, 53% had a favorable outcome within a median observation period of 6.2 months, while 34% of the moderate disease group had a favorable outcome within a median of 5.4 months. However, only 18% of the severe disease group had a favorable outcome within a median observation period of 40.3 months. In addition, the cumulative favorable outcome rate evaluated using Kaplan–Meier curves showed a statistically significant difference between the curves (*p* = 0.003) (Figure 2). There was a tendency for favorable outcomes to occur earlier and more frequently as the BACES severity decreased.

### 3.3. Factors Associated with the Microbiological Outcomes

We conducted multivariate analysis using Cox proportional hazard regression models to assess factors associated with microbiological outcomes in study patients (Table 3). We constructed two analysis models based on two microbiological outcomes, SCC and favorable outcomes, as outcome criteria. In the model based on SCC as the criterion, there was a tendency for higher BACES severity to have a negative impact on SCC, but this was not statistically significant. However, in terms of favorable outcomes, moderate BACES severity (hazard ratio 0.63; 95% confidence interval 0.44–0.91; *p* = 0.013) and severe BACES severity (hazard ratio 0.37; 95% confidence interval 0.16–0.90; *p* = 0.028) showed significantly negative impact compared to mild BACES severity.

## 4. Discussion

We evaluated whether the severity of BACES at the time of MAC-PD diagnosis affected the rate of SCC in MAC-PD patients who underwent watchful waiting without antibiotics. There was a tendency toward a higher SCC rate with a decrease in the severity of BACES, and multivariate analysis showed that higher BACES severity had a negative impact on favorable outcomes. Thus, our data suggest that the rate of SCC depends on the severity of MAC-PD as evaluated via BACES at the time of diagnosis. Therefore, we believe that the use of BACES at the time of diagnosis in MAC-PD patients can assist in establishing further treatment strategies, including watchful waiting.

A major concern of clinicians treating MAC-PD patients is the difficulty in predicting the likelihood of SCC after confirming the MAC-PD diagnosis. In clinical practice, patients are eager to understand the course of their disease, but there is no accurate predictive tool that can provide precise information on the possibility of SCC for each individual patient. One previous study conducted in South Korea to evaluate stable MAC-PD patients identified several factors related to SCC, such as younger age, higher BMI, and a low bacterial burden (negative AFB smear) [6]. However, in real-world clinical practice, it is nearly impossible to base the decision to start antibiotic treatment or choose watchful waiting solely on a single factor for predicting SCC. Furthermore, despite two previous studies including 200 to 400 MAC-PD patients attempting to identify factors associated with SCC, even multivariate analysis has failed to identify significant factors related to SCC in MAC-PD [7,8]. However, in our study, we demonstrated that the BACES severity, which integrates various relevant clinical factors, can assist in predicting the likelihood of SCC in MAC-PD patients. In these contexts, we believe that our current study holds clinical significance.

Because MAC-PD is a multifactorial disease, there have been numerous attempts to identify immunological, environmental, and microbiological factors associated with disease progression or spontaneous improvement [16,17,18,19]. However, to date, a well-validated predictive tool or biomarker for assessing these factors has not been developed. In these circumstances, we believe that the BACES severity, as evaluated by our study, may be useful in medical practice. In other words, for MAC-PD patients with low BACES severity at the time of diagnosis, there is a high likelihood of achieving SCC without antibiotic treatment. Therefore, in cases where there are no symptoms or radiological deterioration, a strategy of watchful waiting for several months without antibiotics can be chosen as the primary approach. This strategy can reduce the medical burden for both patients and clinicians and help prevent unnecessary antibiotic treatment. On the other hand, patients with high BACES severity have a lower likelihood of achieving SCC and require relatively more frequent clinical follow-up. Thus, in cases where there are suspicions of changes in symptoms or radiological deterioration, an antibiotic treatment strategy can be initiated to prevent the acceleration of disease progression. We believe that treatment strategies based on BACES severity can assist in making decisions for MAC-PD patients in medical practice. Additionally, we believe that further studies in different patient cohorts are needed in this regard.

BACES score was originally an indicator of severity based on the mortality of NTM-PD patients [9]. However, according to recent studies, it is also being considered as a predictor of the response to antibiotic treatment [10,11]. In a recent study conducted at a single institution in South Korea, when 992 MAC-PD patients were divided based on BACES severity, the results showed that 85% of BACES mild patients succeeded in antibiotic treatment, while only 61% of BACES severe patients were successful in their treatment [11]. In another study that evaluated 681 NTM-PD patients, the rate of culture conversion after antibiotic treatment was significantly higher in the BACES mild group compared to the BACES severe group (65% vs. 40%) [10]. These findings suggest that the intensity of antibiotic treatment or drug combinations may need to vary depending on the severity of BACES. In other words, as BACES severity increases, the likelihood of drug treatment failure may be relatively higher, and so the consideration of more aggressive agents or adjunctive lung surgery may also be necessary. However, we do not consider BACES to be a perfect tool because it has several limitations. Specifically, among the BACES assessment criteria, age can vary depending on the evaluation time, and the blood level of ESR can also fluctuate due to various other conditions. Furthermore, factors other than BACES may also influence the clinical course of MAC-PD. In particular, a patient’s various underlying conditions, nutritional status, socioeconomic factors, and living environment can have an impact on the disease. Recent literature has suggested that sources of NTM infection may be present in the surrounding environment, and exposure to water at public baths has also been reported to be associated with NTM-PD development [20,21]. Unfortunately, our current study did not consider these various aspects. However, similar to the development of scoring systems for bronchiectasis, which were followed up by modified scoring systems that have proven effective in clinical practice [22,23,24], we believe that there is a need to develop and refine practical clinical tools for MAC-PD to better serve the field.

In our study, we inevitably had to use the definition of a ‘favorable outcome’. The reason for this is that our study focused on the clinical course of MAC-PD patients who did not receive antibiotics. Even without antibiotics, some MAC-PD patients spontaneously improve, and as a result they cannot produce sputum samples for culture testing. In such cases, they cannot provide sputum for culture three times consecutively. Therefore, they may not meet the criteria for culture conversion. However, clinical improvement is obviously observed, and sputum NTM cultures can be negative only twice. This aligns with the concept mentioned in the ‘NTM-NET consensus statement’ referred to as a ‘clinical cure’ [15]. Consequently, to better reflect real-world data, we used an arbitrary definition of a ‘favorable outcome’ in our study. This arbitrary definition is commonly used in NTM-PD research [25]. However, it is believed that there is a need for the development of better indicators to assess microbiological response in patients who are not receiving antibiotic treatment.

Our study had several limitations. Firstly, it was a retrospective study conducted at a single referral center with specialized NTM-PD clinics in South Korea, so our results may not be generalizable. There may have been a patient selection. especially when considering the higher proportion of women, bias. Second, during the study period, MAC-PD patients who had limited information to assess BACES were excluded. Third, patients with mild symptoms may have had irregular follow-up periods, or there could have been variations in the intervals between sputum tests among patients. Lastly, due to the long study period from 2002 to 2016, there is a possibility that clinical practices in managing MAC-PD patients may have changed, and as a result, the study’s findings may not reflect current treatment practices and outcomes. However, the primary antibiotics used for MAC-PD have remained unchanged since the publication of guidelines between 2007 and 2020 [2,3]. Additionally, the strategy of ‘watchful waiting’ after diagnosis has also remained consistent. Therefore, we believe that there has been minimal variation in the treatment approach for patients in our institution. The primary reason for not including patients diagnosed after 2016 in our study is the need for a sufficient follow-up period after the diagnosis of MAC-PD.

In conclusion, our data suggest that the rate of SCC depends on the severity of MAC-PD as evaluated using BACES at the time of diagnosis. A lower BACES severity may be associated with a higher SCC rate in MAC-PD patients. Therefore, we believe that taking BACES severity into account during the watchful waiting period may assist in determining MAC-PD treatment strategies.

## Figures and Tables

**Table 1:** Baseline characteristics of the 373 MAC-PD patients who underwent watchful waiting without antibiotics.

Variables	Total (*n* = 373)	Spontaneous Negative Culture Conversion (*n* = 153)	No Culture Conversion (*n* = 220)	*p*-Value
BACES items				
BMI < 18.5 kg/m^2^	73 (20)	30 (20)	43 (20)	0.988
Age ≥ 65 years	153 (41)	47 (31)	106 (48)	0.001
Cavity	36 (10)	9 (6)	27 (12)	0.040
Elevated ESR *	234 (63)	96 (63)	138 (63)	0.997
Sex, male	145 (39)	56 (37)	89 (41)	0.453
Ex- or current smoker	98 (26)	113 (74)	161 (73)	0.885
Underlying condition				
Previous tuberculosis	103 (28)	43 (28)	60 (27)	0.860
Chronic obstructive pulmonary disease	24 (6)	16 (11)	8 (4)	0.008
Chronic pulmonary aspergillosis	2 (1)	-	-	0.515
Lung cancer	18 (5)	6 (4)	12 (6)	0.497
Symptoms				
Cough	230 (62)	99 (65)	131 (60)	0.313
Sputum	227 (61)	94 (61)	133 (61)	0.848
Hemoptysis	63 (17)	28 (18)	35 (16)	0.544
Weight loss	17 (5)	4 (3)	13 (6)	
Positive sputum AFB smear	122 (33)	40 (26)	82 (37)	0.024
Etiology				0.081
*M. avium*	229 (61)	102 (67)	127 (58)	
*M. intracellulare*	144 (39)	51 (33)	93 (42)	
Radiological phenotype				0.096
Nodular bronchiectatic form	353 (94)	147 (96)	206 (94)	
Fibrocavitary form	20 (5)	6 (4)	14 (6)	

Data are presented as number (%) or median (interquartile range). BACES, BMI, age, cavity, ESR, and sex; BMI, body mass index; ESR, erythrocyte sedimentation rate; AFB, acid-fast bacilli. * Elevated ESR: >15 mm/h in men and >20 mm/h in women.

**Table 2:** Microbiological outcomes of patients who underwent watchful waiting without antibiotics according to BACES severity.

Watchful Waiting	Total (*n* = 373)	Mild (*n* = 183)	Moderate (*n* = 157)	Severe (*n* = 33)	*p*-Value	*p-*Trend ^†^
Spontaneous negative culture conversion	153 (41)	87/183 (48)	58/157 (37)	8/33 (24)	0.017 ^ac^	0.005
Time to culture conversion, months	6.2 (2.7–17.7)	6.0 (2.2–16.1)	5.4 (2.8–17.2)	22.1 (14.2–88.5)	0.008 ^bc^	0.037
Favorable outcome *	157 (42)	97/183 (53)	54/157 (34)	6/33 (18)	<0.001 ^ac^	<0.001
Time to favorable outcome, months	6.2 (2.5–15.8)	6.2 (2.3–14.3)	5.4 (2.5–14.0)	40.3 (11.8–98.2)	0.022 ^bc^	0.346

Data are presented as number (%) or median (interquartile range). BACES, BMI, age, cavity, ESR, and sex; BMI, body mass index; ESR, erythrocyte sedimentation rate. * Maintenance of culture conversion or having two consecutive negative sputum cultures without any further positive culture results until the last follow-up date. ^†^ *p* for trend was obtained using a linear-by-linear method and Jonckheere–Terpstra test. ^a^ *p* < 0.05 with Bonferroni correction between mild and moderate groups. ^b^ *p* < 0.05 with Bonferroni correction between moderate and severe groups. ^c^ *p* < 0.05 with Bonferroni correction between mild and severe groups.

**Table 3:** Factors associated with microbiological outcome in patients who underwent watchful waiting without antibiotics (*n* = 373).

Variables	Univariable Analysis		Multivariable Analysis	
	Unadjusted HR (95% CI)	*p*-Value	Adjusted HR (95% CI)	*p*-Value
**Spontaneous negative culture conversion**				
Ex- or current smoker	0.85 (0.59–1.22)	0.388	0.99 (0.66–1.49)	0.971
Underlying condition				
Previous pulmonary tuberculosis	1.02 (0.72–1.46)	0.901	1.12 (0.77–1.62)	0.556
Chronic obstructive pulmonary disease	1.54 (0.91–2.58)	0.106	1.76 (1.02–3.05)	0.043
Lung cancer	0.92 (0.40–2.07)	0.915	0.89 (0.38–2.08)	0.792
Etiology				
*M. avium*	Reference		Reference	
*M. intracellulare*	0.83 (0.60–1.17)	0.287	0.83 (0.59–1.17)	0.295
BACES severity				
Mild	Reference		Reference	
Moderate	0.75 (0.54–1.05)	0.093	0.74 (0.52–1.07)	0.106
Severe	0.53 (0.26–1.10)	0.089	0.49 (0.22–1.08)	0.076
Positive sputum AFB smear at diagnosis	0.68 (0.47–0.97)	0.034	0.70 (0.48–1.01)	0.057
Favorable outcome *				
Ex- or current smoker	0.77 (0.53–1.12)	0.167	1.04 (0.69–1.57)	0.861
Underlying condition				
Previous pulmonary tuberculosis	0.83 (0.58–1.19)	0.313	0.95 (0.65–1.38)	0.767
Chronic obstructive pulmonary disease	1.11 (0.63–1.95)	0.728	1.35 (0.75–2.44)	0.317
Lung cancer	0.89 (0.40–2.02)	0.785	1.01 (0.44–2.33)	0.982
Etiology				
*M. avium*	Reference		Reference	
*M. intracellulare*	0.67 (0.48–0.94)	0.021	0.67 (0.47–0.94)	0.020
BACES severity				
Mild	Reference		Reference	
Moderate	0.63 (0.45–0.88)	0.007	0.63 (0.44–0.91)	0.013
Severe	0.37 (0.16–0.84)	0.017	0.37 (0.16–0.90)	0.028
Positive sputum AFB smear	0.79 (0.55–1.12)	0.787	0.84 (0.59–1.20)	0.333

HR, hazard ratio; CI, confidence interval; BACES, BMI, age, cavity, ESR, and sex; BMI, body mass index; ESR, erythrocyte sedimentation rate; AFB, acid-fast bacilli. * Maintenance of culture conversion or having two consecutive negative sputum cultures without any further positive culture results until the last follow-up date.

## Data Availability

All relevant data are within the paper.

## Supplementary Materials

The following supporting information can be downloaded at: https://www.mdpi.com/article/10.3390/jcm12227125/s1, Figure S1: Cumulative culture conversion rate in all study patients according to BACES severity.
