# Supplementary material for: Spontaneous Cultural Conversion Rate of Mycobacterium avium Complex Pulmonary Disease Based on BACES Severity
Source: J Clin Med. 2023 Nov 16;12(22):7125. doi: 10.3390/jcm12227125 (PMC10671925; doi:10.3390/jcm12227125)
Supplement: Supplementary file 1 [file jcm-12-07125-s001.zip › jcm-2712338-supplementary.pdf]

Supplementary material

Spontaneous Cultural Conversion Rate of *Mycobacterium avium* complex pulmonary disease Based on BACES severity

Bo-Guen Kim[1#], Jin Young Yu[2#], Byung Woo Jhun[3]

[1]Division of Pulmonary Medicine and Allergy, Department of Internal Medicine, Hanyang University College of Medicine, Seoul, South Korea.

[2]Department of Medicine, Samsung Medical Center, Sungkyunkwan University School of Medicine, Seoul, South Korea

[3]Division of Pulmonary and Critical Care Medicine, Department of Medicine, Samsung Medical Center, Sungkyunkwan University School of Medicine, Seoul, South Korea.

[#]Bo-Guen Kim and Jin Young Yu contributed equally to this work

Correspondence: Byung Woo Jhun, Division of Pulmonary and Critical Care Medicine, Department of Medicine, Samsung Medical Center, Sungkyunkwan University School of Medicine, 81 Irwon-ro, Gangnam-gu, Seoul 06351, Republic of Korea. E-mail: byungwoo.jhun@gmail.com

Supplementary figure 1. Cumulative culture conversion rate in all study patients according to BACES severity

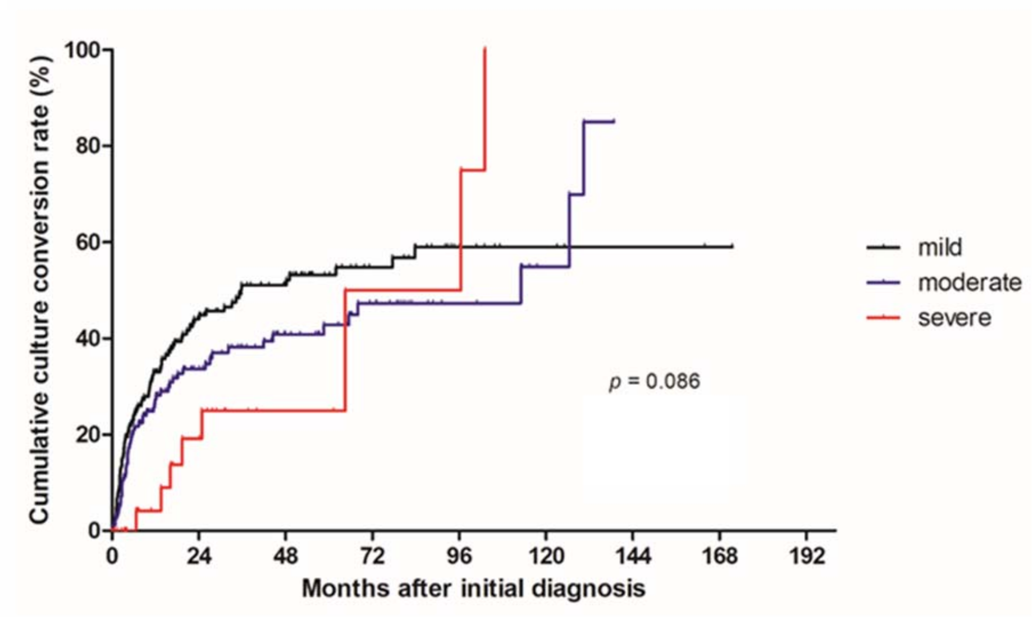

No. at risk								
**BACES Mild**	183	69	46	23	11	4	2	1
**BACES Moderate**	157	64	35	21	8	3	0	0
**BACES Severe**	33	15	4	2	2	0	0	0
